# Surgical Treatment of a Large Post-cesarean Hematoma in the Retzius Space Accompanied by a Blood Coagulation Disorder: A Rare Case Report

**DOI:** 10.7759/cureus.51417

**Published:** 2023-12-31

**Authors:** Anna Thanasa, Efthymia Thanasa, Vasiliki Grapsidi, Emmanouil M Xydias, Ektoras-Evangelos Gerokostas, Ioannis Rafail Antoniou, Evangelos Kamaretsos, Apostolos C Ziogas, Ioannis Paraoulakis, Ioannis Thanasas

**Affiliations:** 1 Department of Health Sciences, Aristotle University of Thessaloniki, School of Medicine, Thessaloniki, GRC; 2 Department of Obstetrics and Gynecology, General Hospital of Trikala, Trikala, GRC; 3 Department of Obstetrics and Gynecology, EmbryoClinic IVF, Thessaloniki, GRC; 4 Department of Obstetrics and Gynecology, University of Thessaly, Larissa, GRC

**Keywords:** hematoma, retzius space, caesarean section, blood coagulation disorder, ultrasound, ct scan, percutaneous puncture, re-operation, case report

## Abstract

Hematoma in the Retzius space after a cesarean section is a rare complication. The Retzius space, also referred to as the prevesical or retropubic space, represents an extraperitoneal artificial cavity situated between the pubic symphysis and the bladder. In instances where conservative treatment involving vigilant monitoring along with analgesics and antibiotics or ultrasound-guided percutaneous puncture proves unsuccessful, re-operation becomes imperative. Our case report concerns a second-parity pregnant patient who underwent a cesarean section in the 39th gestational week. A decrease in hemoglobin level on the third postoperative day, combined with the onset of febrile infection, an increase in inflammatory markers, and the manifestation of lower abdominal pain, prompted a thorough investigation of the puerperant. Imaging revealed the existence of a hematoma in the Retzius space associated with a mild blood coagulation disorder. Subsequently, the unsuccessful outcome of the ultrasound-guided percutaneous puncture of the hematoma, combined with the persistence of clinico-laboratory findings, led to the decision to perform a re-laparotomy on the 10th postoperative day after the cesarean section. During the surgery, a large hematoma was identified in the Retzius space, extending below the rectus abdominis muscles. The procedure involved surgical drainage of the hematoma, meticulous hemostasis, and the placement of negative pressure drainage in the Retzius space. The patient was discharged from the clinic on the fifth postoperative day after re-operation. Ten days later, both blood tests and ultrasounds were without abnormal findings. In this paper, following the case presentation, a brief review is provided regarding the diagnostic and therapeutic approach of patients with hematoma in the Retzius space after cesarean section.

## Introduction

Complications following a cesarean section are more prevalent compared to vaginal delivery. In contemporary obstetrics, the increasing frequency of cesarean sections is driven not only by medical necessities but also by medico-legal and ethical considerations [1], including cases initiated at the maternal request without a specific medical indication. Based on recent literature data, it is estimated that in those cases where the pregnant woman insists on her refusal to induce labor and there are no clinical indications for a cesarean section, the obstetrician-gynecologist must respect the choice of the pregnant woman. Consequently, the rates of complications post-cesarean section have increased in recent years [1]. Hematoma in the space of Retzius is a rare complication after cesarean section or other gynecological surgeries, which was first described by Schiffer and Hellman in 1970 [2]. The Retzius space, also referred to as the prevesical or retropubic space, represents an extraperitoneal artificial cavity situated between the pubic symphysis and the bladder. This cavity is formed at the boundary between the transversalis fascia of the abdominal wall and the parietal peritoneum [3,4]. The development of hematoma in the Retzius space after cesarean section can be attributed to the extensive vascularization of the anatomic area, particularly when combined with inadequate hemostasis, the intense separating of the rectus abdominis muscles, and suboptimal abdominal wall suturing technique [5]. Less commonly, the hematoma may extend into the extraperitoneal space between the rectus abdominis muscles, as in our patient, and cause varying degrees of blood coagulation disorder in the puerperant.

The case reports a rare condition, the potential benefits of conservative treatment, including percutaneous puncture, as well as the importance of prompt and optimal surgical intervention, which contributes significantly to reducing maternal morbidity and mortality rates. At the same time, it should be emphasized that the correlation between hematoma in the prevesical space and blood coagulation disorders, even if mild, demands serious consideration when monitoring puerperants display signs of hemodynamic instability and intra-abdominal infection in the immediate post-operative period after a cesarean section.

## Case presentation

Our case concerns a 30-year-old, second-parity pregnant woman. With an obstetric history of two prior cesarean sections, she delivered her third child via an elective cesarean section at the 39th gestational week. Her personal medical history was unremarkable, and she received prenatal care at the gynecology outpatient clinic of the General Hospital of Trikala. The pregnancy was uncomplicated with normal results from obstetric ultrasonography (first-trimester ultrasound, second-level ultrasound, and Doppler), a regular glucose challenge test, and a negative vaginal culture for group B Streptococcus. Preoperative blood tests revealed no abnormal findings (Table 1). The patient had no family history of bleeding or clotting disorders. The patient was not tested for coagulation disorders.

**Table 1:** Preoperative and postoperative laboratory analysis of a patient with hematoma after cesarean section at the Retzius space Hemodynamic instability is evident, which manifested on the third postoperative day, accompanied by a mild blood coagulation disorder due to the formation of a large hematoma in the Retzius space. CS: cesarean section; R/L: relaparotomy; HT: hematocrit; HB: hemoglobin; PLT: platelets; WBC: white blood cells; NEUT: neutral; CRP: C reactive protein; APTT: activated partial thromboplastin time; INR: international normalized ratio; FIB: fibrinogen; U: urea; CR: creatinine; B: bilirubin; SGOT: serum glutamic oxaloacetic transaminase; SGPT: serum glutamate pyruvate transaminase

Laboratory tests	Preoperatively	1st Postoperative day after CS	3rd Postoperative day after CS	4th Postoperative day after CS	6th Postoperative day after CS	9th Postoperative day after CS	10th Postoperative day after R/L	Normal laboratory values
Ht	33%	32%	23.8%	28.3%	27.7%	31.2%	33.6%	37.7 – 49.7%
HB	11.4 gr/dl	10.9 gr/dl	7.9 gr/dl	9.4 gr/dl	9.2 gr/dl	10.5 gr/dl	10.9 gr/dl	11.8 – 17.8 gr/dl
PLT	311x10^3^/ml	341x10^3^/ml	173x10^3^/ml	165x10^3^/ml	163x10^3^/ml	170x10^3^/ml	210x10^3^/ml	150 – 350 x10^3^/ml
WBC	11.2x10^3^/ml	12.9x10^3^/ml	14.9x10^3^/ml	16.5x10^3^/ml	17.8x10^3^/ml	18.1x10^3^/ml	8.5x10^3^/ml	4 – 10.8 x10^3^/ml
NEUT	75.9%	84.3%	78.5%	82.2%	81%	79.8%	54%	40 – 75%
CRP	0.49 mg/dl		9.8 mg/dl	17.1 mg/dl	21.1 mg/dl	25.3 mg/dl	0.81 mg/dl	<0.7 mg/dl
APTT	32.2 sec		39.2 sec	39.7 sec	39.6 sec	39.5 sec	34.5 sec	24.0 – 35.0 sec
INR	1.01		1.21	1.29	1.28	1.27	1.07	0.8 – 1.2
FIB	516 mg/dl		708 mg/dl	710 mg/dl	690 mg/dl	673 mg/dl	462 mg/dl	200 – 400 mg/dl
U	12 mg/dl		22 mg/dl		31 mg/dl	46 mg/dl	17 mg/dl	10 – 50 mg/dl
CR	0.40 mg/dl		0.51 mg/dl		0.41 mg/dl	0.47 mg/dl	0.51 mg/dl	0.40 – 1.10 mg/dl
B			0.24 mg/dl		0.14 mg/dl	0.45 mg/dl	0.43 mg/dl	0.3 – 1.2 mg/dl
SGOT			24 IU/L		15 IU/L	36 IU/L	20 IU/L	5 – 33 IU/L
SGPT			22 IU/L		18 IU/L	44 IU/L	34 IU/L	10 – 37 IU/L

Intraoperatively, the Kerr incision on the uterine wall was sutured, and meticulous hemostasis was maintained in the anatomic area of the vesicouterine pouch. There were no adhesions, the surgery was uneventful, and there was no bleeding during abdominal wall closure. Closure of the abdominal wall included diligent hemostasis in both the prevesical space and the rectus abdominis muscles.

**Figure 1:**
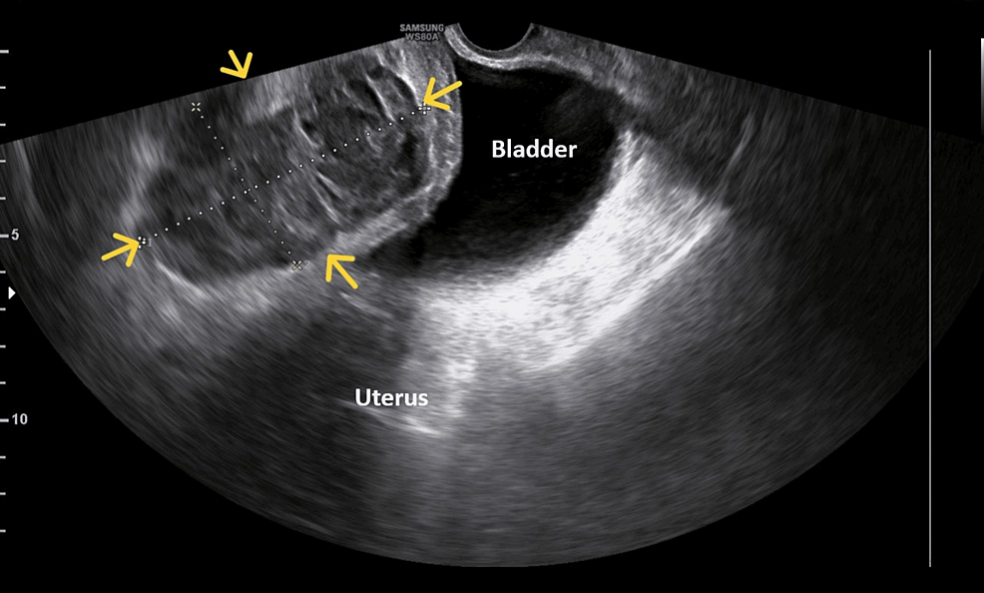
Transvaginal ultrasound image of a hematoma in the Retzius space after a cesarean section The presence of a well-circumscribed cystic lesion (yellow arrows) is evident, exerting pressure on the thickened bladder wall.

After surgery, the patient's hemodynamic status was stable, with a blood pressure of 120/70 mmHg and a pulse rate of 90 bpm. The pain responded to mild painkillers. The decline in hemoglobin levels accompanied by a mild blood coagulation disorder, as revealed by the patient's blood tests on the third postoperative day (Table 1), combined with the onset of fever (up to 38^O^C), the increase in inflammation markers, and the manifestation of lower abdominal pain, prompted the decision for further evaluation of the puerperant through pelvic imaging. Transvaginal ultrasound revealed extensive fluid collection of mixed echogenicity with solid components in the prevesical space, estimated to be approximately 12x8cm (Figure 1).

**Figure 2:**
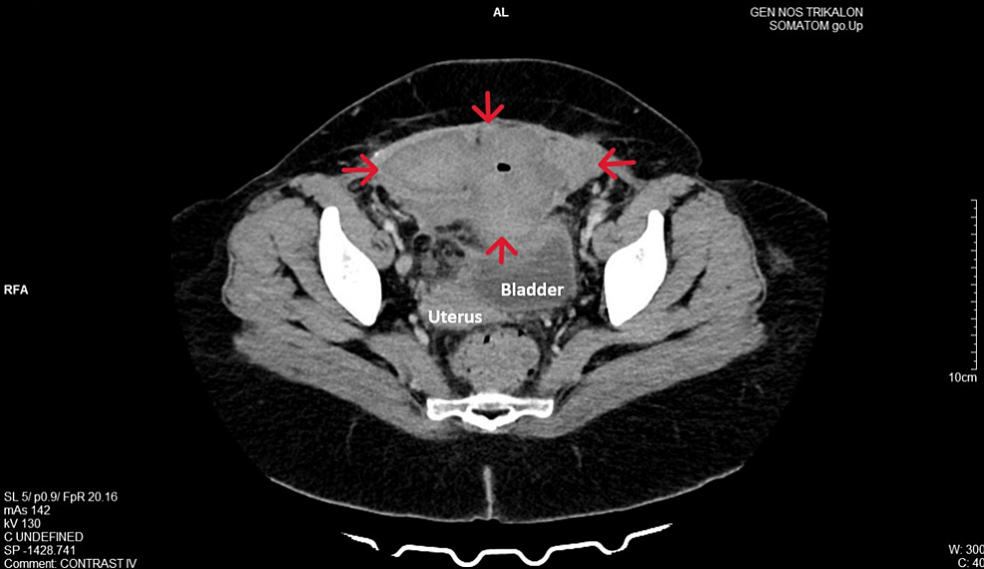
CT image of a hematoma in the Retzius space after cesarean section The presence of an encapsulated fluid collection is noted, depicting a large organized hematoma (red arrows) located on the anterior abdominal wall.

Computed tomography subsequently confirmed these ultrasound findings, identifying an encapsulated fluid collection in the Retzius space as an enlarged organized hematoma (15.5x8.5x8.5cm). This hematoma was situated on the anterior abdominal wall, exerting pressure on the superior surface of the bladder (Figure 2). The patient had no symptoms of urinary retention or anuria.

After transfusing the patient with two units of packed red blood cells and two units of fresh frozen plasma, the patient's hemodynamic status was stabilized. Laparotomy was not chosen at that time, as it was thought that waiting might lead to resorption of the hematoma. Also, vascular embolization was not available in our hospital. Renal and liver function tests were within the normal range (Table 1). A culture of lochia and blood culture were received, which were negative for growth.

**Figure 3:**
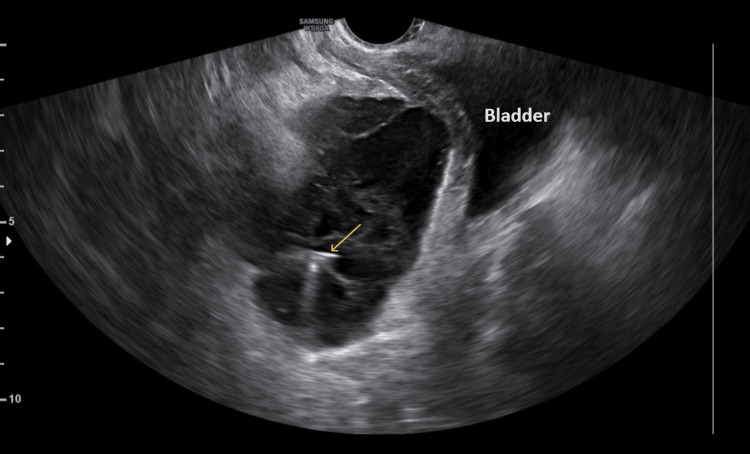
Ultrasound-guided percutaneous puncture of a hematoma at the Retzius space after a cesarean section After easy insertion of the puncture needle (yellow arrow) into the hematoma cavity, drainage of the collection proved to be unfeasible.

Initially, for the treatment of the hematoma, a systematic administration of analgesics and broad-spectrum antibiotics was decided. Due to non-improvement in the clinico-laboratory findings, on the eighth postoperative day, it was decided to proceed with the ultrasound-guided percutaneous puncture of the hematoma from the prevesical space, which however proved unfeasible (Figure 3).

**Figure 4:**
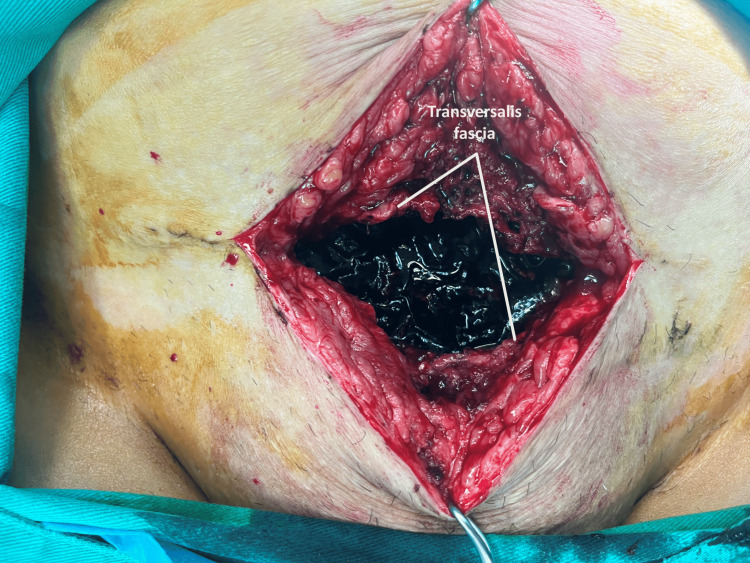
Intraoperative image of hematoma in the Retzius space after cesarean section Following the opening of the transversalis fascia, multiple blood clots are evident in the subfascial space between the rectus abdominis muscles.

The persistence of the patient's symptoms without improvement of inflammation markers and the unchanged imaging of the fluid collection in the Retzius space, as revealed by a repeat computed tomography scan performed the following day (ninth postoperative day), led to the decision to proceed with laparotomy on the 10th postoperative day after the cesarean section. Intraoperatively, a large hematoma was identified in the Retzius space, extending to the rectus abdominis muscles (Figure 4).

**Figure 5:**
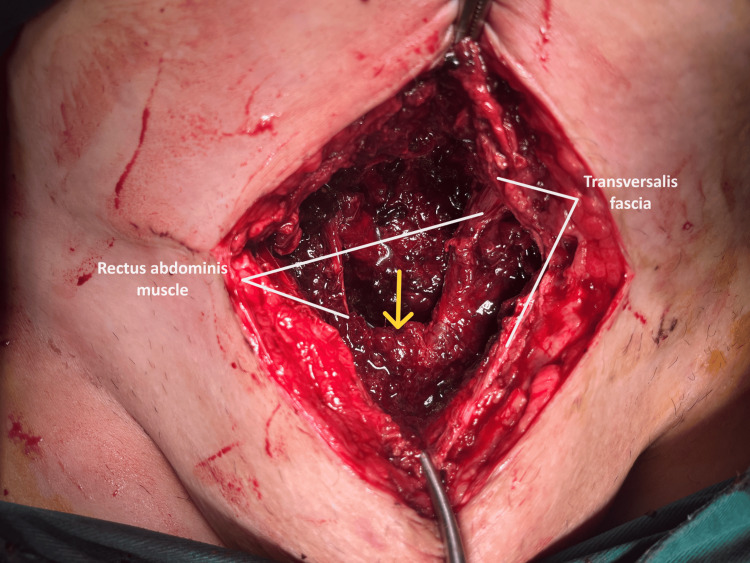
Intraoperative image of a hematoma in the Retzius space after a cesarean section Following the evacuation of the hematoma cavity from the blood clots, the rectus abdominis muscles and the Retzius space are demarcated (yellow arrow).

Drainage of the blood clots and thorough drainage of the hematoma cavity were carried out (Figure 5).

The anterior bladder wall appeared thickened due to the delayed drainage of the hematoma. To confirm the integrity of the bladder, 300 ml of normal saline solution was administered through the Foley catheter with clear urine output. Meticulous hemostasis was then ensured, followed by the placement of negative pressure drainage in the Retzius space.

**Figure 6:**
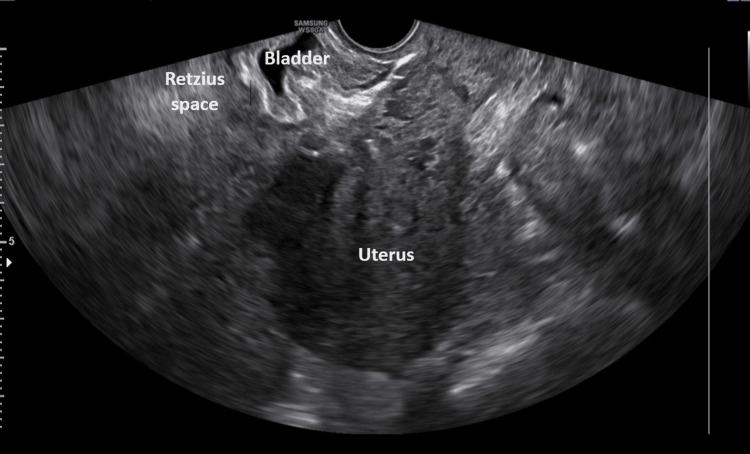
Postoperative ultrasound image Following surgical drainage of the hematoma in the Retzius space after cesarean section, no fluid collection is observed posterior to the rectus abdominal muscles in the prevesical space.

Immediately after re-operation, remission of fever, gradual improvement of inflammation markers, and blood coagulation (activated partial thromboplastin time (APTT), international normalized ratio (INR)) were observed. The patient was discharged from the clinic on the 14th postoperative day after a cesarean section with consultation for re-examination at the gynecological outpatient clinic. Ten days later, blood tests (Table 1) and ultrasound imaging of the prevesical space (Figure 6) revealed no abnormal findings.

## Discussion

The hematoma in the Retzius space, and by extension, the subfascial hematoma, is an extraperitoneal collection of blood located in the prevesical space and posterior to the rectus abdominis muscles. It is crucial to distinguish the hematoma in the Retzius space from the bladder flap hematoma, which is situated in the intraperitoneal cavity between the bladder and the lower segment of the uterus, as well as from the superficial-wound hematoma [6,7]. The diagnosis of hematoma in the Retzius space following a cesarean section cannot rely solely on clinical signs [8]. Lower abdominal pain, accompanied by signs of hemodynamic instability, is the predominant symptom in these patients. However, it's important to note that lower abdominal pain, particularly when of mild to moderate intensity and occurring in the initial hours post-cesarean section and not accompanied by signs of hypovolemia (such as nausea, dizziness, vomiting, or tachycardia), can easily be attributed to the surgical procedure and postpartum uterine activity. In our patient, a decline in hemoglobin levels compared with the preoperative level and values from the first postoperative day was observed in the blood tests conducted on the third postoperative day after the cesarean section (Table 1). The decrease in hemoglobin value was accompanied only by mild, non-evaluable lower abdominal pain along the surgical site.

In contrast to the clinical signs, the contribution of imaging to the diagnosis of hematoma in the Retzius space is significant. Ultrasound (transabdominal or transvaginal), as a readily available examination without the use of ionizing radiation, is the primary imaging modality in the diagnostic evaluation of subfascial hematoma. Ultrasound typically reveals a cystic or mixed-echogenicity complex mass located posterior to the rectus abdominis muscles and anterior to the bladder, within the prevesical space. The size of the hematoma can vary significantly. The presence of air bubbles within the hematoma may be an indication of hematoma infection and abscess formation [9]. In certain instances, confirming the diagnosis of hematoma in the Retzius space may require computed tomography with or without angiography. Computed tomography with contrast can also be valuable in detecting contrast medium extravasation, indicating active bleeding [5,6]. In our patient, both transvaginal ultrasound and transabdominal ultrasound, along with computed tomography, successfully diagnosed the formation of hematoma in the Retzius space, excluding cases of bladder flap hematoma and superficial-wound hematoma. The air bubble detected during the repeat computed tomography on the ninth postoperative day was accurately attributed to the attempted percutaneous puncture of the hematoma rather than indicating infection.

Surgical drainage of the hematoma from the Retzius space seems to be the preferred approach, especially in cases of large hematomas accompanied by deterioration of the clinical condition, despite attempts at conservative management. Conservative management, involving analgesics, antibiotics, and regular, meticulous clinical monitoring, along with imaging evaluation, is recommended for asymptomatic patients or those with mild symptoms. The administration of analgesics and broad-spectrum antibiotics is considered to contribute significantly to preventing infection and providing supportive care until hematoma absorption, which, depending on its size, can take from days to weeks [10]. Before deciding on re-operation and surgical drainage of the hematoma, attempting ultrasound-guided percutaneous puncture of the hematoma could be considered a secondary step following conservative management of these patients [8]. In our patient, persistent clinical symptoms and a continued increase in inflammation markers, without significant improvement in the imaging of the hematoma on computed tomography and ultrasound, led to the decision to drain the hematoma. We initially attempted ultrasound-guided percutaneous puncture of the hematoma, but the procedure was unsuccessful. The presence of blood clots and organized components within the hematoma cavity were the primary factors contributing to the failure of the procedure. Consequently, re-operation and surgical drainage of the hematoma were the only viable options. Intraoperatively, the presence of hematoma in the Retzius space was confirmed by extension between the rectus abdominis muscles, and drainage was performed (Figure 4, Figure 5) without requiring peritoneal opening and entry into the peritoneal cavity.

## Conclusions

Hematoma in the Retzius space after cesarean section, especially when particularly associated with a blood coagulation disorder, is a rare postpartum complication. Hematoma in the Retzius space should be considered in the differential diagnosis of puerperants displaying signs of intra-abdominal infection with hemodynamic instability in the immediate post-operative period after cesarean section. Timely diagnosis, accurate evaluation, and optimal management are crucial to reducing maternal morbidity and mortality.
